# Dutch guideline for clinical foetal-neonatal and paediatric post-mortem radiology, including a review of literature

**DOI:** 10.1007/s00431-018-3135-9

**Published:** 2018-04-19

**Authors:** L. J. P. Sonnemans, M. E. M. Vester, E. E. M. Kolsteren, J. J. H. M. Erwich, P. G. J. Nikkels, P. A. M. Kint, R. R. van Rijn, W. M. Klein, On behalf of the Dutch post-mortem imaging guideline group, W. L. J. M. Duijst, P. A. M. Hofman, J. J. F. Kroll, N. S. Renken, Y. O. Rosier, C. I. E. Scheeren, S. J. Stomp, P. van der Valk

**Affiliations:** 1 Department of Radiology, Radboud University Medical Center, Nijmegen, The Netherlands; 2 Department of Radiology, Academic Medical Center, Amsterdam, The Netherlands; 3 Department of Forensic Medicine, Netherlands Forensic Institute, The Hague, The Netherlands; 4 Amsterdam Centre for Forensic Science and Medicine, Amsterdam, The Netherlands; 5 Knowledge Institute of Medical Specialists, Utrecht, The Netherlands; 6 Department of Obstetrics and Gynaecology, University Medical Center Groningen, University of Groningen, Groningen, The Netherlands; 7 Department of Pathology, University Medical Center Utrecht, Utrecht, The Netherlands; 8 Department of Radiology, Amphia Hospital, Breda, The Netherlands; 9 Department of Radiology, Maastricht University Medical Center, Maastricht, The Netherlands

**Keywords:** Post-mortem, Paediatric, Neonatal, Foetal, Radiology, Autopsy

## Abstract

Clinical post-mortem radiology is a relatively new field of expertise and not common practice in most hospitals yet. With the declining numbers of autopsies and increasing demand for quality control of clinical care, post-mortem radiology can offer a solution, or at least be complementary. A working group consisting of radiologists, pathologists and other clinical medical specialists reviewed and evaluated the literature on the diagnostic value of post-mortem conventional radiography (CR), ultrasonography, computed tomography (PMCT), magnetic resonance imaging (PMMRI), and minimally invasive autopsy (MIA). Evidence tables were built and subsequently a Dutch national evidence-based guideline for post-mortem radiology was developed. We present this evaluation of the radiological modalities in a clinical post-mortem setting, including MIA, as well as the recently published Dutch guidelines for post-mortem radiology in foetuses, neonates, and children. In general, for post-mortem radiology modalities, PMMRI is the modality of choice in foetuses, neonates, and infants, whereas PMCT is advised in older children. There is a limited role for post-mortem CR and ultrasonography. In most cases, conventional autopsy will remain the diagnostic method of choice.

*Conclusion*: Based on a literature review and clinical expertise, an evidence-based guideline was developed for post-mortem radiology of foetal, neonatal, and paediatric patients.

**Table:** 

**What is Known:**
*• Post-mortem investigations serve as a quality check for the provided health care and are important for reliable epidemiological registration.*
*• Post-mortem radiology, sometimes combined with minimally invasive techniques, is considered as an adjunct or alternative to autopsy.*
**What is New:**
*• We present the Dutch guidelines for post-mortem radiology in foetuses, neonates and children.*
*• Autopsy remains the reference standard, however minimal invasive autopsy with a skeletal survey, post-mortem computed tomography, or post-mortem magnetic resonance imaging can be complementary thereof.*

## Introduction

Paediatric post-mortem radiology, in addition to autopsy, is becoming widely accepted as an important component of cause of death determination [1–5]. The trend in declining clinical autopsy rates in adults [6–9] is also evident in the foetal and paediatric population, though higher autopsy rates of approximately 50% remain [3, 10–13]. This decline is in spite of evidence that clinical error rates persist: approximately 25% discrepancy between clinical ante-mortem diagnosis and autopsy cause of death diagnosis [3, 11, 14, 15].

If an alternative, less- or non-invasive diagnostic method could adequately determine the cause of death, current objections to conventional autopsy (e.g. its invasiveness) could be met. Consequently, this might increase quality control and subsequently improve clinical care. Post-mortem radiology might be such an alternative diagnostic method. It can be helpful for diagnosing anatomic abnormalities, identification of syndromes, or to narrow down the differential diagnosis of genetic disorders. Consequently, it can also be useful for identifying potential siblings at risk, counselling for future pregnancies, and helping the parents in their process of grief [7, 16].

Post-mortem radiology is evolving into a subspecialty, reflected by the large increase of publications and the broad spectrum of used techniques [1]. Nevertheless, a guideline on the indications and contraindications for the use of post-mortem conventional radiography (CR), ultrasonography, computed tomography (PMCT), magnetic resonance imaging (PMMRI), and minimally invasive autopsy (MIA), was not yet available. This article provides the literature review that is the basis for the evidence-based Dutch guideline for clinical foetal, neonatal, and paediatric post-mortem radiology [17].

## Materials and methods

The guideline was developed under the guidance of the Dutch knowledge institute of medical specialists. An important objective of the Dutch knowledge institute is to preserve and pool knowledge and expertise about the design and execution of quality assurance projects in the realm of specialist medical care. Medline and Embase were searched for studies comparing clinical post-mortem radiology to autopsy in foetal, neonatal, and paediatric patients from January 2000 up to January 2016, when the guideline committee started her work (Appendix 1, a further detailed search strategy is available upon request). Language selection was restricted to studies published in Dutch and English. The study selection and analysis was performed separately for the group of foetal and neonatal cases (deceased within 28 days post-partum) and for the group of paediatric cases (aged 1 month to 18 years). Studies were initially screened on title and abstract (JE, RR), and hereafter analysed on full text (EK, JE, RR). Case reports and forensic articles were excluded. Outcomes in sensitivity and specificity were mandatory. Reference lists of included studies were screened for additional relevant studies.

Methodological quality assessment of included studies was performed (EK) according to the AMSTAR checklist, PRISMA checklist, or QUADAS II, depending on the type of article [18–20]. The joint evidence of included articles was scored (EK) according to the Grading Recommendations Assessment, Development and Evaluation (GRADE) tool [21]. GRADE divides the quality (or certainty) of evidence and conclusions into four categories: high, medium, low or very low. A high GRADE level of evidence means that the conclusion is unlikely to change with future research, whereas in a very low GRADE level of evidence the conclusion is very precarious. In addition to the level of evidence in literature, expertise from the Dutch post-mortem imaging guideline group members was taken into account, along with preferences of bereaved relatives, costs, availability of devices, and organisational issues when formulating the guideline recommendations.

## Results

### Study identification

**Fig. 1:**
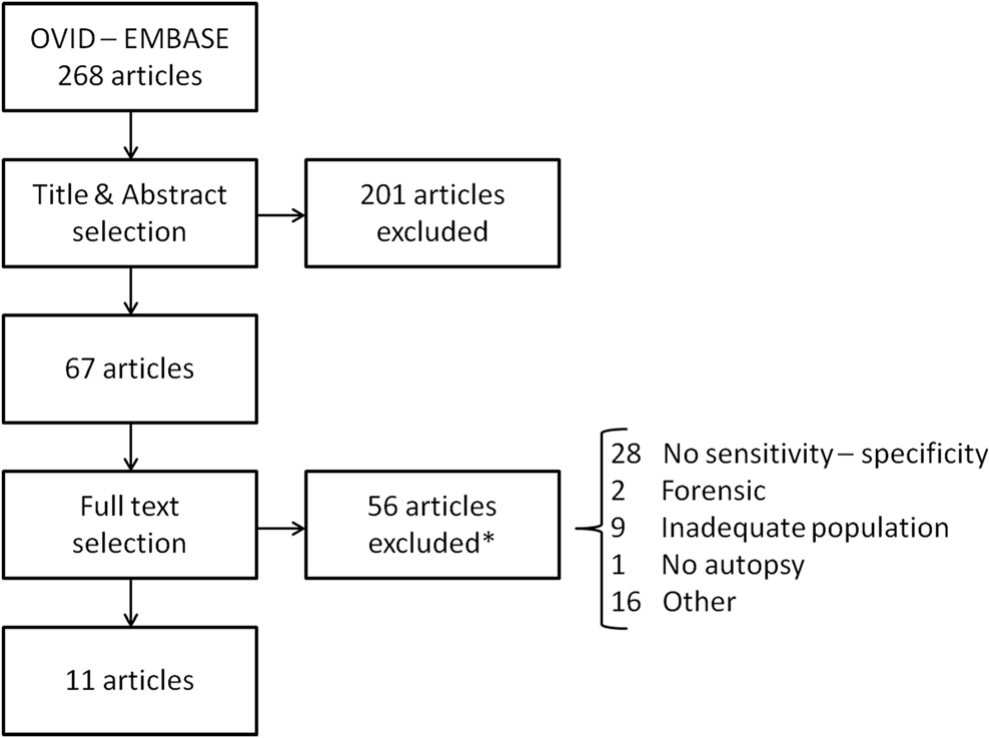
Foetal-neonatal study selection. * Several papers had multiple reasons for full text exclusion. Maximum one reason per article was scored, according to the order presented

**Fig. 2:**
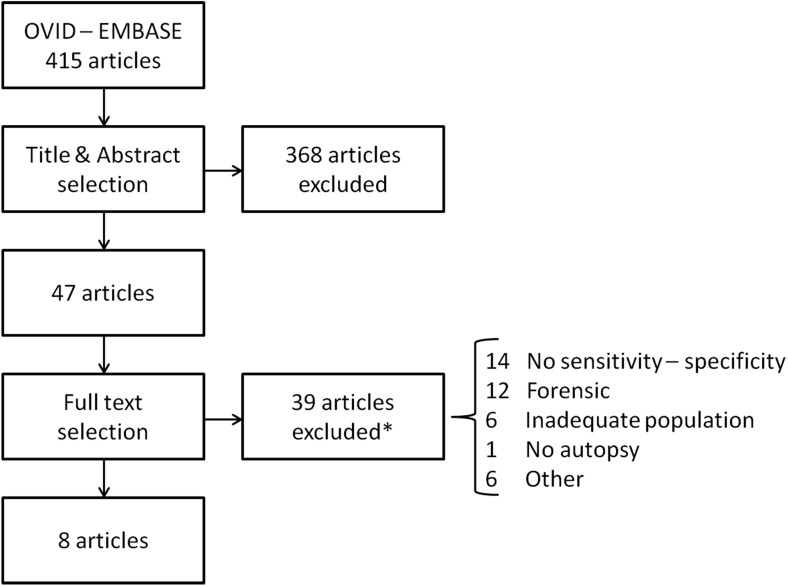
Paediatric study selection. * Several papers had multiple reasons for full text exclusion. Maximum one reason per article was scored, according to the order presented

The literature search resulted in 268 eligible articles for foetuses and neonates and 415 articles for paediatric studies. After title, abstract, and full-text selection 14 foetal-neonatal articles and 9 paediatric studies remained (Figs. 1 and 2). Studies on CR, PMCT, PMMRI, and MIA were included, other post-mortem imaging methods (e.g. post-mortem ultrasound) did not meet the inclusion criteria.

### Study quality

Both in foetal and neonatal patients, as well as in paediatric patients, the GRADE evidence for post-mortem CR, PMCT, and MIA was graded as low because few studies, with few patients included, have been performed. More studies were published on PMMRI, yet the evidence for PMMRI was also graded as low in both groups, because almost all results were based on the Magnetic Resonance Imaging Autopsy Study (MaRIAS). This study was performed in a specialised setting with well-trained specialists and a relatively low number of patients.

### Post-mortem conventional radiography (CR)

One article was included from the literature review on post-mortem CR in foetal and neonatal patients. No articles on post-mortem CR of paediatric patients met the inclusion criteria.

#### Foetal-neonatal

**Fig. 3:**
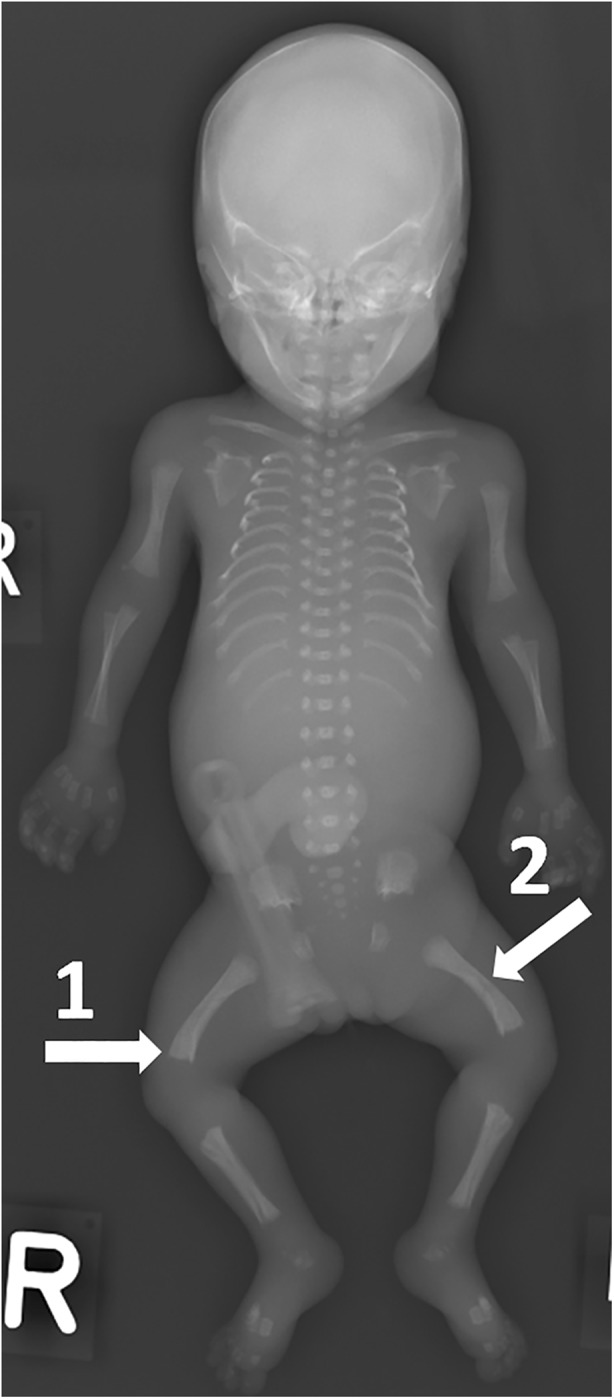
Example of a diagnostic babygram. This pregnancy was terminated at 22 weeks of gestation because of micromelia on prenatal 2nd trimester ultrasound, suspected to be a skeletal dysplasia. The babygram showed skeletal abnormalities with shortened ribs, metaphyseal flaring (1) and shortened and bowed long bones (2). Histology revealed abnormalities in the liver, kidneys, lungs, bone and cartilage compatible with ciliopathy with major skeletal involvement. Jeune syndrome is the most likely diagnosis

A clinical, foetal post-mortem skeletal survey consists mainly of a whole-body radiograph; a ‘babygram’ (Fig. 3). A study of 377 foetal post-mortem skeletal surveys showed a 100% sensitivity and 97% specificity for detection of skeletal abnormalities compared to diagnosis based on autopsy, genetics, and prenatal investigations [22]. However, the number of diagnostic abnormalities in this population was limited. The authors concluded that there is no indication for a foetal post-mortem skeletal survey in cases without previous suspicion of skeletal abnormalities on prenatal ultrasound or during post-mortem external inspection. If a foetal ‘babygram’ is obtained, it should preferably be done using a high resolution ‘cabinet radiography’ system. If this is not available, the use of a mammography system is advised.

### Post-mortem computed tomography (PMCT)

One study was included on the sensitivity and specificity of PMCT for both foetal-neonatal and paediatric patients, and an additional article on paediatric patients (Table 1).

**Table 1:** Table of evidence of diagnostic performance of PMCT in foetuses, neonates and paediatric patients

Author (ref)	Year	Study design	Anatomical system	Outcome parameter	Foetuses < 24 weeks of gestation		Foetuses > 24 weeks of gestation		Neonates and children	
					Sensitivity (95%CI)	Specificity (95%CI)	Sensitivity (95%CI)	Specificity (95%CI)	Sensitivity (95%CI)	Specificity (95%CI)
Arthurs [23]	2016	Prospective (MaRIAS)	General	Main diagnosis or cause of death	28 (13–51)	100 (44–100)	50 (22–79)	100 (61–100)	50 (29–71)	100 (74–100)
				Any lesion (cardiac, thoracic, neurologic, abdominal or skeletal)	67 (35–88)	91 (81–96)	62 (36–82)	93 (80–97)	58 (42–72)	95 (89–98)
			Cardiac	Cardiac lesions	NA	100 (61–100)	100 (21–100)	100 (65–100)	50 (22–79)	100 (83–100)
			Non-cardiac thoracic	Non-cardiac thoracic lesions	0 (0–79)	100 (51–100)	100 (34–100)	80 (38–96)	60 (36–80)	85 (58–96)
			Neurologic	Neurologic lesions	100 (44–100)	67 (44–84)	50 (22–79)	100 (57–100)	58 (32–81)	100 (82–100)
			Abdominal	Abdominal lesions	0 (0–79)	100 (65–100)	100 (21–100)	67 (30–90)	50 (10–91)	93 (77–98)
			Musculo-skeletal	Skeletal lesions	75 (30–95)	100 (89–100)	0 (0–79)	100 (82–100)	100 (21–100)	96 (82–99)
Arthurs [24]	2015	Prospective	Non-cardiac thoracic	Abnormal lung areas					100 (52–100)^a^	63% (31–86)^a^

^a^Using ventilated PMCT

#### Foetal-neonatal

PMCT and PMMRI were both compared to autopsy, and to each other in 53 foetuses, finding 40% of the PMCT’s non-diagnostic in foetuses below 24 weeks of gestation (*n* = 35) compared to 11% of PMMRI’s, with twice as many correct diagnoses on PMMRI compared to PMCT (10 vs. 5, *p* < 0.005) [23]. In foetuses above 24 weeks of gestation (*n* = 18), 22% of PMCT’s were non-diagnostic, compared to 0% of PMMRI’s (*p* < 0.005). In cases where radiology was diagnostic, both PMCT and PMMRI showed a 50% sensitivity and 100% specificity for main diagnosis or cause of death. Also, no significant differences were observed for identification of pathological lesions in individual organ systems, irrespective of contribution to death.

#### Paediatric

Sensitivity of PMCT for cause of death determination depends on the type of pathology and age of the child [23, 24]. The same study as for foetuses and neonates, included 29 children with an average age of 6.9 months (range 1 day–16 years) [23]. In this small group, both PMCT and PMMRI showed a 50% sensitivity and 100% specificity for the main diagnosis or cause of death. The overall concordance was slightly lower for PMCT than PMMR (59.4% vs. 62.8%). In another study with 12 children under the age of 1 year, PMCT’s of the lungs were non-diagnostic in 75% prior to post-mortem ventilation, compared to 0% of PMCT’s with ventilation [24]. A 100% sensitivity and 63% specificity were found for the detection of abnormal lung areas with ventilated PMCT. Therefore, ventilated PMCT could be used to improve identification of abnormal areas of the lungs.

### Post-mortem magnetic resonance imaging (PMMRI)

Seven articles were included on the diagnostic performance of PMMRI in foetuses and neonates, along with five articles on paediatric patients (Table 2). The majority of these studies reported on the Magnetic Resonance Imaging Autopsy Study (MaRIAS) (sub)population [23, 25, 27–30]. MaRIAS is a large, 3.5 year, double-blind prospective study in 277 foetuses (185 foetuses of 24 weeks gestation or less and 92 foetuses of 24 weeks gestation or more) and 123 children (42 neonates, 53 infants up to 1 year of age, and 28 children above 1 year of age), which compared the diagnostic accuracy of 1.5 T PMMRI to conventional autopsy [31, 32].

**Table 2:** Table of evidence of diagnostic performance of PMMRI in foetuses, neonates and paediatric patients

Author (ref)	Year	Study design	Anatomical system	Outcome parameter	Foetuses < 24 weeks of gestation		Foetuses > 24 weeks of gestation		Neonates and children	
					Sensitivity (95%CI)	Specificity (95%CI)	Sensitivity (95%CI)	Specificity (95%CI)	Sensitivity (95%CI)	Specificity (95%CI)
Arthurs [23]	2016	Prospective (MaRIAS)	General	Main diagnosis or cause of death	40 (23–59)	100 (61–100)	50 (24–76)	100 (68–100)	50 (29–71)	100 (74–100)
				Any lesion (cardiac, thoracic, neurologic, abdominal or skeletal)	58 (39–76)	88 (81–93)	79 (57–92)	92 (83–96)	70 (55–82)	84 (76–90)
			Cardio-vascular	Cardiac lesions	100 (44–100)	86 (69–94)	100 (44–100)	100 (80–100)	63 (31–86)	95 (77–99)
			Non-cardiac thoracic	Non-cardiac thoracic lesions	20 (4–62)	96 (78–99)	67 (21–94)	87 (62–96)	47 (26–69)	50 (25–75)
			Neurological	Neurologic lesions	83 (44–97)	52 (32–72)	75 (41–93)	70 (40–89)	100 (76–100)	77 (53–90)
			Abdominal	Abdominal lesions	40 (12–77)	100 (86–100)	100 (51–100)	93 (69–99)	100 (34–100)	82 (63–92)
			Musculo-skeletal	Skeletal lesions	60 (23–88)	100 (88–100)	0 (0–79)	100 (82–100)	100 (21–100)	96 (82–99)
Taylor [25]	2014	Prospective (MaRIAS)	Cardio-vascular	Structural and non-structural heart diseases	82 (59–94)	96 (91–98)	83 (44–97)	94 (87–97)	62 (41–79)	98 (93–100)
				Structural heart defects (major and minor)	83 (61–94)	97 (92–99)	100 (57–100)	93 (85–98)	100 (77–100)	98 (94–100)
				Major structural heart defects	87 (62–96)	99 (95–100)	100 (51–100)	99 (94–100)	100 (68–100)	100 (97–100)
Votino [26]	2012	Prospective (High-field MRI, 9.4 T)	Cardio-vascular	Abnormalities of the four-chamber view	67 (30–92)	80 (52–95)				
				Outflow-tract-abnormalities	75 (20–96)	100 (83–100)				
				Abnormalities of the aortic arch	100^a^	100^a^				
				Abnormalities of the systemic veins	100^a^	100^a^				
Arthurs [27]	2014	Prospective (MaRIAS)	Non-cardiac thoracic	Non-cardiac thoracic abnormalities	30 (17–47)	96 (91–98)	38 (19–61)	88 (79–94)	45 (33–58)	61 (48–72)
Arthurs [28]	2015	Prospective (MaRIAS)	Neurological	Ischaemic brain injury	NA	100 (97–100)	30 (11–60)	90 (81–95)	93 (70–99)	95 (89–98)
				Major intracranial bleed	100 (21–100)	99 (95–97)	100 (21–100)	100 (96–100)	100 (82–100)	99 (95–100)
				Minor intracranial bleed	100 (21–100)	87 (80–91)	80 (38–96)	99 (93–100)	100 (44–100)	98 (94–100)
				Brain malformations	86 (69–94)	90 (83–94)	90 (60–98)	96 (87–99)	100 (57–100)	100 (97–100)
				Overall brain pathology	87 (71–95)	69 (60–77)	71 (53–84)	77 (64–86)	98 (90–100)	81 (70–89)
Arthurs [29]	2015	Prospective (MaRIAS)	Abdominal	Abdominal abnormalities	77 (61–88)	95 (90–98)	65 (41–83)	89 (80–95)	71 (47–87)	87 (79–92)
Arthurs [30]	2014	Prospective (MaRIAS)	Musculo-skeletal	Skeletal abnormalities	69 (50–84)	100 (97–100)	17 (3–56)	98 (92–99)	31 (13–58)	96 (91–99)

^a^When visualisation was possible

#### Foetal-neonatal

Before the MaRIAS study, a systematic review, investigating the diagnostic accuracy of PMMRI, included five studies on foetuses [33]. In four of those five studies, a complete autopsy was used as the reference standard. The included studies were of moderate quality as the groups were small and the population heterogeneity large. There was a pooled sensitivity of 69% (95%CI 56–80) and a pooled specificity of 95% (95%CI 88–98) for detection of clinically significant abnormalities.

The MaRIAS study reported high sensitivities (82–100%) and high specificities (93–97%) for both major and minor cardiac pathology, as well as for structural and non-structural heart disease in foetuses below and above 24 weeks of gestation [25]. Votino et al. (2012) compared high-field PMMRI (9.4 T) to lower-field PMMRI (1.5 T and 3.0 T) and stereomicroscopic autopsy (MIA) [26]. In contrast to lower-field PMMRI, the heart situs, four-chamber view and outflow tracts could be visualised in all foetuses with 9.4 T, irrespective of gestational age. High-field PMMRI identified seven out of eight cases with major congenital heart disease. In foetuses below and above 24 weeks of gestation, MaRIAS reported low sensitivities of 30 and 38% and high specificities of 96 and 88% respectively for the detection of non-cardiac, thoracic abnormalities with 1.5 T [27]. Based on these results and the reasonable negative predictive values of approximately 85%, PMMRI appeared to be more useful in the exclusion of thoracic abnormalities, rather than in its identification. Detection of pulmonary tract infection and diffuse alveolar haemorrhage was difficult, whereas PMMRI was most sensitive for detection of anatomical abnormalities, including pleural effusions and lung hypoplasia.

**Fig. 4:**
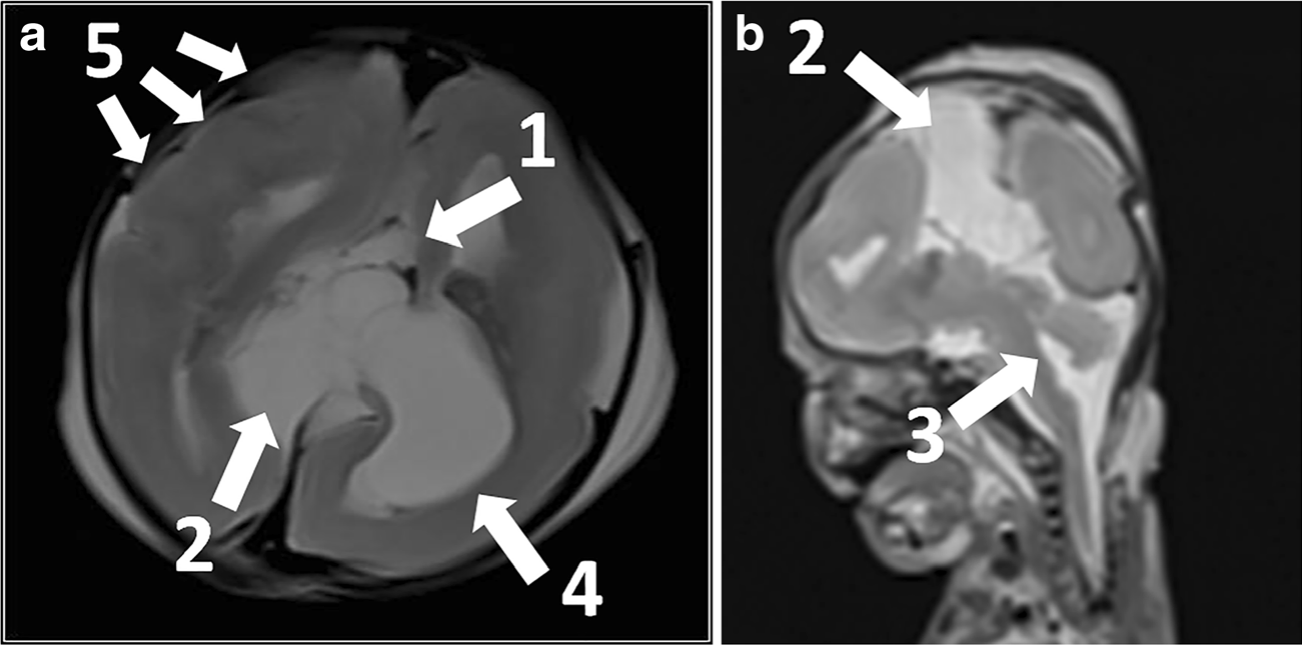
**a**, **b** Example of abnormalities of the central nervous system diagnosed at PMMRI in a female foetus. This pregnancy was terminated at 23 weeks of gestation because of corpus callosum agenesis (1), an interhemispheric cyst (2) and fossa posterior anomalies on prenatal 2nd trimester ultrasound, which were confirmed by PMMRI and/or conventional autopsy. A non-cystic dilatation of the fourth ventricle (3) was found on PMMRI along with the additional findings of a left choroid plexus cyst (4) and polymicrogyria (5). Furthermore, autopsy diagnosed a choroid plexus papilloma in the left lateral ventricle, but the additional finding of polymicrogyria (5) on PMMRI revealed Aicardi syndrome as the most likely diagnosis. **a** Axial. **b** Sagittal

Based on MaRIAS, very high sensitivities (80–100%) and specificities (87–100%) were found for the detection of brain malformations (Fig. 4) and minor and major intracranial bleedings [28]. A lower sensitivity of 30% was found for the detection of hypoxic-ischaemic brain injury in foetuses above 24 weeks of gestation. Furthermore, cerebral PMMRI provided clinically important information in 23 out of 43 foetuses in whom neuropathological examination was non-diagnostic due to maceration.

PMMRI showed moderate sensitivities of 77 and 65% for abdominal abnormalities in foetuses below and above 24 weeks gestation, respectively [29]. Diagnostic accuracy was variable per organ system, with the highest sensitivity for renal abnormalities (18/21 = 86%) and the lowest for intestinal abnormalities (2/7 = 29%). In addition, MaRIAS reported moderate and very low sensitivities for detection of musculoskeletal abnormalities in foetuses below and over 24 weeks of gestation, respectively 69 and 17% [30].

#### Paediatric

MaRIAS reported a 100% sensitivity and 98% specificity for major and minor structural heart defects in neonates and children with 1.5 T PMMRI [25]. A substantial lower sensitivity of 62% was observed for any cardiac pathology, both structural and non-structural. Identification of non-cardiac, thoracic abnormalities was difficult, especially in case of pneumonia [27]. Sensitivities of 100% and specificities of 98–100% were reported for the detection of brain malformations and minor and major intracranial haemorrhages [28]. In contrast to foetuses, PMMRI showed a high sensitivity (93%) for ischaemic brain injury in neonates and children. Just as in foetuses, PMMRI showed a moderate sensitivity (71%) and high specificity (87%) for abdominal abnormalities [29]. The sensitivity for skeletal abnormalities was poor (31%) [30].

### Minimal invasive autopsy (MIA)

One article included from the literature search reported on MIA in both foetal-neonatal and paediatric patients (MaRIAS) [31]. This study compared the diagnostic accuracy of MIA to conventional autopsy. MIA consisted of PMMRI, combined with other post-mortem radiology, genetic and metabolic tests (ante-mortem and post-mortem blood sampling), a review of the clinical history, external examination, and examination of placental tissue, if available. No foetal-neonatal or paediatric studies combining PMMRI or PMCT with tissue biopsies or angiography met the inclusion criteria.

#### Foetal-neonatal

Both a high sensitivity (100%, 95%CI 97–100) and high specificity (98%, 95%CI 88–100) were reported for the detection of major pathological abnormalities or cause of death in foetuses below 24 weeks of gestation [31]. In foetuses above 24 weeks of gestation, sensitivity and specificity were also high (respectively 96%, 95%CI 86–99, and 95%, 95%CI 84–99). Moreover, MIA had a higher sensitivity and specificity compared to PMMRI alone. In both groups of foetuses, the sensitivity and specificity for detection of non-infectious pathologies were above 95%. Sensitivity for infectious pathologies was with 80% (95%CI 38–96) lower in foetuses above 24 weeks of gestation than in foetuses below 24 weeks of gestation (100%, 95%CI 92–100).

#### Paediatric

A 69% sensitivity (95%CI 58–78) and 93% specificity (95%CI 81–98) were found for major pathological abnormalities or cause of death in children [31]. Sensitivities of respectively 94% (95%CI 84–98) and 27% (95%CI 14–44) were reported for the detection of non-infectious and infectious pathologies, with specificities of respectively 96% (95%CI 89–99) and 100% (95%CI 96–100). Pneumonia and myocarditis were the main undetected abnormalities. This study showed an increase in the diagnostic accuracy of post-mortem radiology when PMMRI was extended with additional (minimal-invasive, genetic and metabolic) tests or examination of placental tissue. Like in the foetal patient group, MIA showed better results than PMMRI alone.

### Dutch post-mortem imaging guideline

**Fig. 5:**
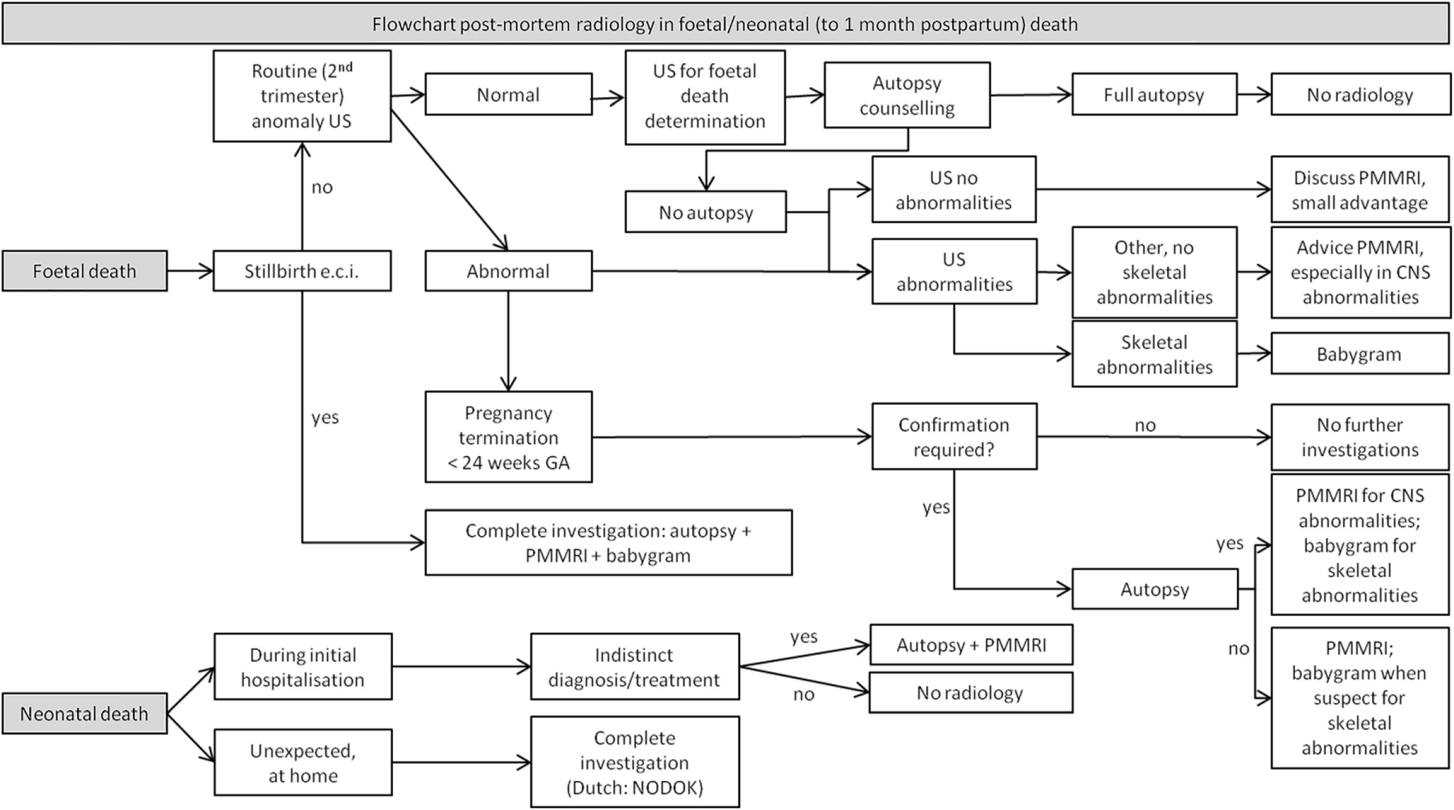
Flowchart for post-mortem radiology in foetal and neonatal deaths*. * adapted from the Dutch guideline for clinical foetal, neonatal, and paediatric post-mortem radiology [17]. GA: gestational age. US: ultrasonography. The ‘routine 2^nd^ trimester ultrasound’ is a standard prenatal US in all growing foetuses. The ‘US for foetal death determination’ is a second, separate antenatal US by the gynaecologist in order to confirm death. PMMRI: post-mortem magnetic resonance imaging. CNS: central nervous system. NODOK:: The Dutch ‘Nader Onderzoek naar de DoodsOorzaak van Kinderen’ (i.e. ‘further examination of cause of death in children’) procedure is a stepwise approach to investigate the cause of death in children with an assumed natural unexpected and unexplained death [34]

**Fig. 6:**
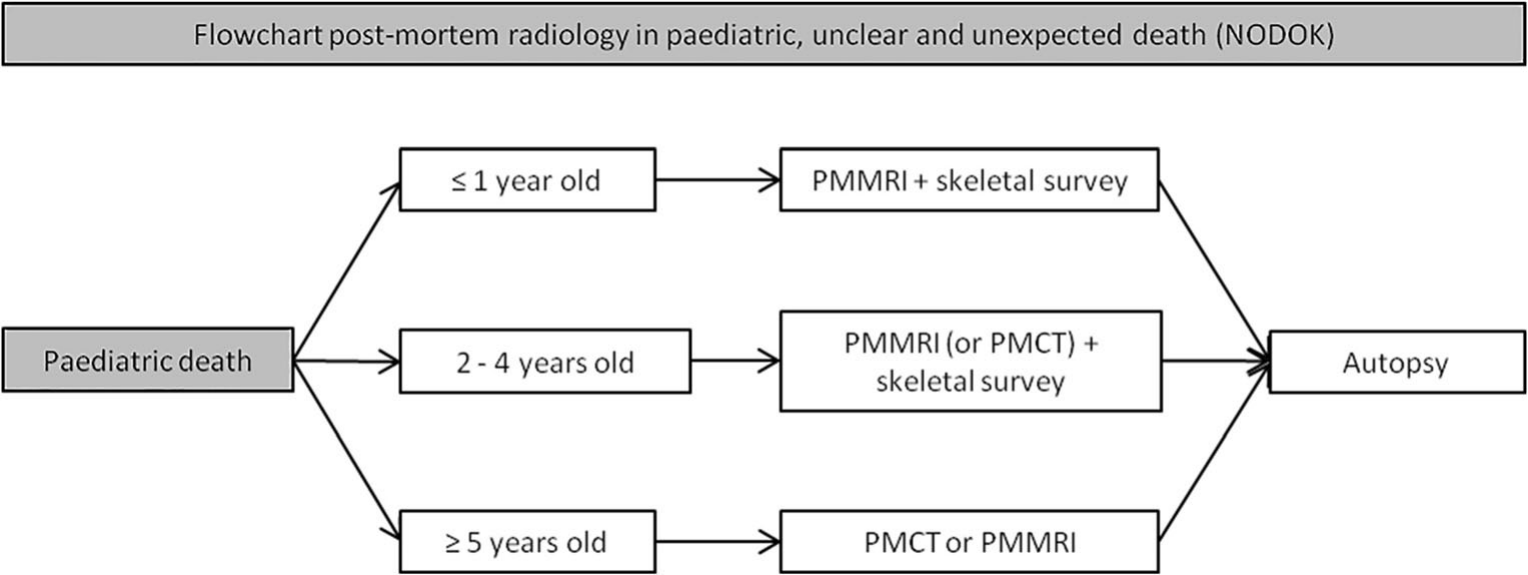
Flowchart for post-mortem radiology in paediatric deaths*. * adapted from the Dutch guideline for clinical foetal, neonatal, and paediatric post-mortem radiology [17]. PMMRI: post-mortem magnetic resonance imaging. PMCT: post-mortem computed tomography. NODOK: The Dutch ‘Nader Onderzoek naar de DoodsOorzaak van Kinderen’ (i.e. ‘further examination of cause of death in children’) procedure is a stepwise approach to investigate the cause of death in children with an assumed natural unexpected and unexplained death [34]

The Dutch guideline working group developed an evidence and practice-based flowchart for post-mortem radiology in non-forensic foetal and neonatal deaths (Fig. 5), and paediatric deaths (Fig. 6). It must be emphasised that, based on the literature, due to the low GRADE level of evidence, post-mortem radiology without clinical autopsy should be considered as insufficient for best-practice post-mortem diagnosis.

## Discussion

**Fig. 7:**
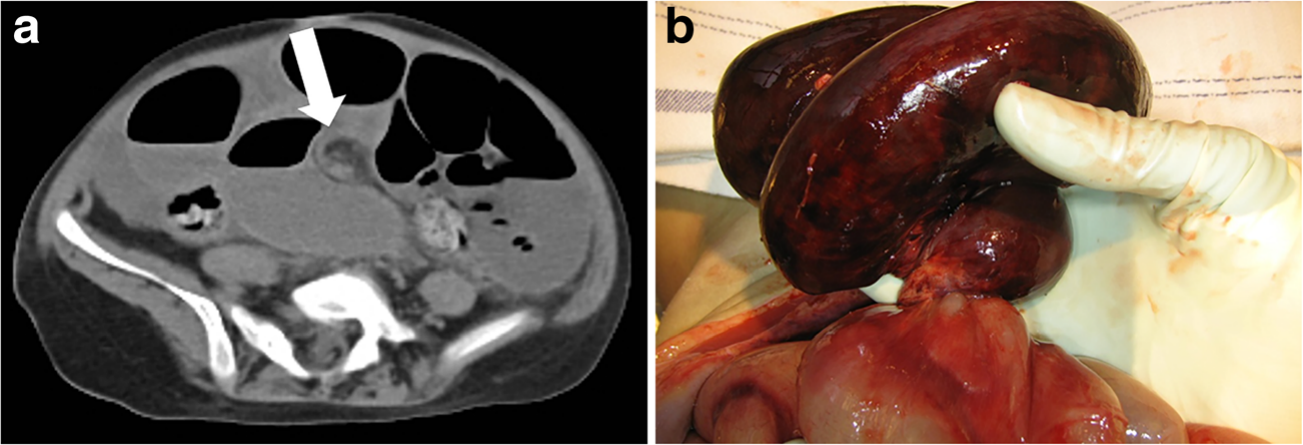
**a**, **b** Example of a PMCT (**a**) of a 4-year-old child with an unexpected and unexplained but assumed natural cause of death. Cardiopulmonary resuscitation was performed but not successful. PMCT and PMMRI showed a volvulus of the ileum around its mesentery (whirl sign) (arrow). Ischemic haemorrhagic volvulus of the ileum was confirmed by autopsy (**b**) as the cause of death

Autopsy is traditionally considered as the gold standard for post-mortem diagnoses and quality assessment of provided health care. However, the declining autopsy rates of the last decennia result in decreasing expertise, especially in foetal-neonatal and paediatric cases where mortality rates are low. Although autopsy remains the preferred diagnostic method in foetal, neonatal, and paediatric death, post-mortem radiology, after consent, can be used in adjunct to autopsy or as an alternative in cases without consent for conventional autopsy. In general, PMMRI is advised in foetuses, neonates, and young children, as PMMRI has a higher soft-tissue contrast compared to PMCT. The small body size enables high-resolution whole-body imaging in a reasonable amount of time. The limited value of PMCT in young children is illustrated in a study of 54 children (median age 1.0 years old, range 2 days–17.9 years) who died of an assumed natural cause, where PMCT could establish the cause of death in mere 12.9% [34]. In older children, just as in adults, PMCT is the preferred modality because of the lack of evidence of superiority of PMMRI over PMCT, its high availability, lower costs, and reduced scan time compared to PMMRI. With the limited amount of studies in children, it is not possible to be more specific about the age range where PMCT and PMMRI have equal diagnostic performances. The Dutch guideline for paediatric post-mortem radiology describes PMCT as a possible adjunct to PMMRI and autopsy, in children of 2 to 5 years of age (Fig. 7). Furthermore, either PMCT or PMMRI is advised in children of 5 years or older, depending on the type of pathology expected. Given the limited amount of evidence, we would like to underline that, especially in infants and children, post-mortem imaging should be seen as an adjunct to the autopsy and not as a replacement. The cut-off age levels were the results of combined expert opinion, this as there is insufficient evidence to define a set cut-off age level.

No eligible paediatric studies on post-mortem CR were included. Nevertheless, in deceased children up to 4 years of age, a skeletal survey (consisting of 20–30 images) is advised to detect fractures, potentially caused by non-accidental injury [35–39]. In deceased children of 5 years or older, with possible child abuse, conventional radiographs of the areas of interest are advised on a low-threshold basis. This is despite a lack of evidence for the supplementary value of a skeletal survey or conventional radiographs in natural causes of death. In a study in 542 perinatal deaths (from 16 weeks gestation to 1 week after birth), the diagnostic value was very limited: 30% had abnormal radiographs, of which only 0.9% were of diagnostic importance for establishing the cause of death [40].

Although ultrasound did not meet the inclusion criteria for the guideline it is a technique that could be considered in selected cases where parents do not approve the use of PMCT or PMMRI [41–43]. Due to open sutures and absence of inhaled air, the brain and lungs can be examined by ultrasonography in cases of foetal demise [44]. In 88 foetuses of 11–40 weeks of gestation sensitivities of 91, 88, and 87% and specificities of respectively 90, 92, and 95% were reported with ultrasound for respectively brain, thoracic and abdominal anomalies [45].

**Fig. 8:**
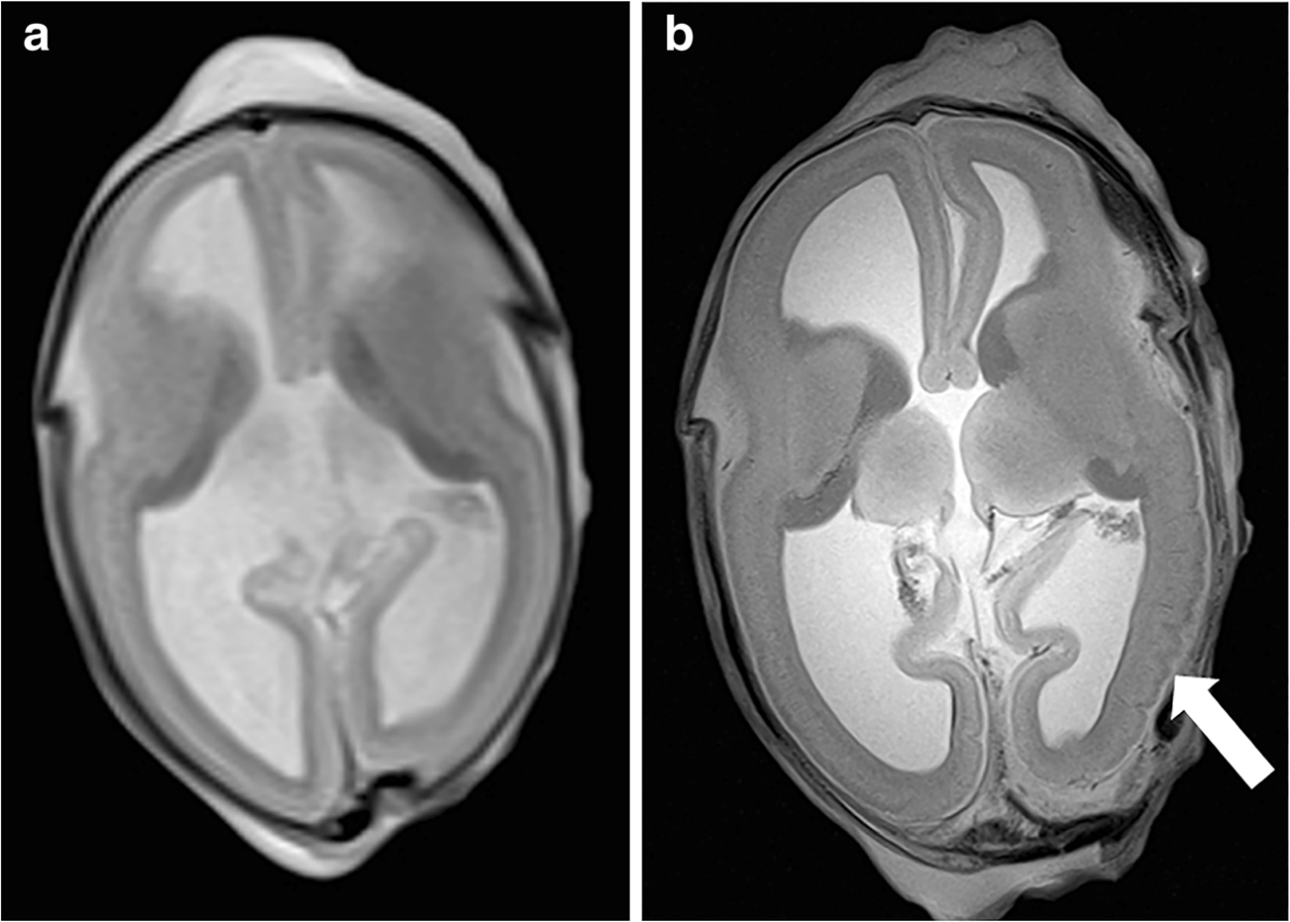
**a**, **b** Example of the difference in resolution between 1.5 (**a**) and 7 (**b**) Tesla PMMRI in a foetus of 18 weeks and 2 days of gestation. The 7 T image shows development of polymicrogyria (arrow) of the left temporal cortex, which is not detectable at the 1.5 T images

To meet the demand for less invasive alternatives to autopsy [46, 47], as well as a high diagnostic performance, it is likely that a combination of imaging and minimal invasive tissue acquisition will be increasingly used in future. Other minimal invasive techniques such as genetic and metabolic testing as well as virology and microbiology sampling can be added on indication. The more post-mortem radiology is expanded with minimally invasive investigations, the higher the diagnostic yield will be; the border area of a minimal invasive radiological test and a restricted autopsy demands for close collaboration between these two specialities. Furthermore, the diagnostic performance of post-mortem radiology will increase by improvements of diagnostic techniques such a high-field PMMRI (Fig. 8), post-mortem angiography, and post-mortem ventilation [24, 48, 49]. Non- or minimally invasive autopsy evokes much less objections from parents compared to conventional autopsy, resulting in overall increasing post-mortem investigation rates [46, 47]. Hence, post-mortem radiology can increase post-mortem investigation rates, and subsequently improve family counselling and quality control of clinical diagnosis.

**Fig. 9:**
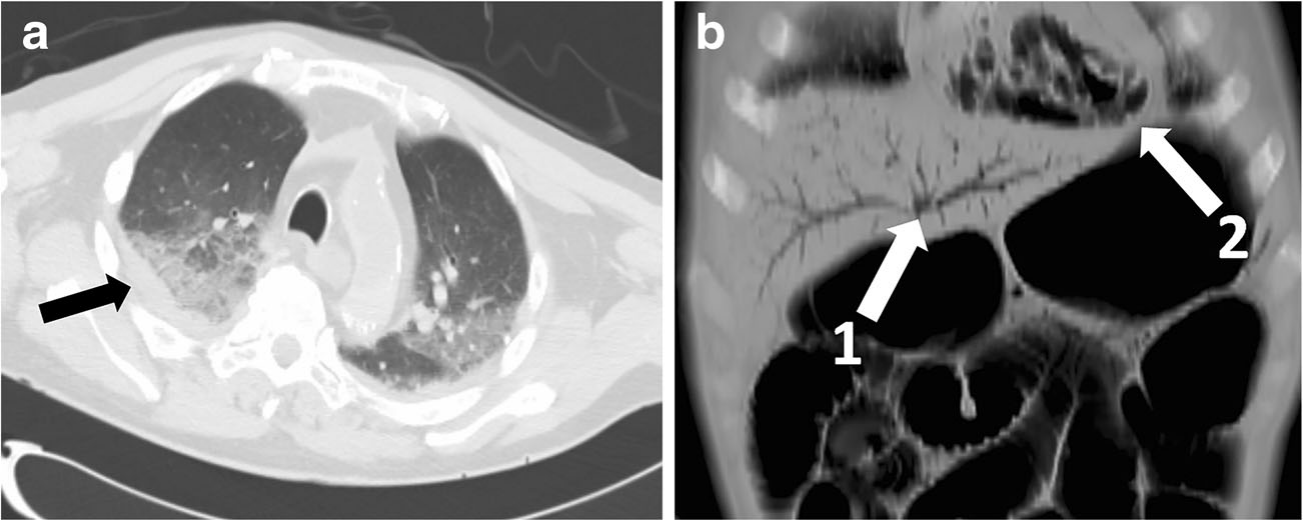
**a**, **b** Examples of normal post-mortem findings. (**a**) Opacification dorsal in the lung lobes due to septal oedema and pleural fluid (arrow). (**b**) Distension of bowel lumen due to post-mortem gas formation, and portal venous (1) and ventricular gas (2)

As post-mortem radiology is a relatively new subspecialty, images should be evaluated by an experienced radiologist. This should preferably be a paediatric radiologist who is familiar with normal post-mortem changes, which to the untrained eye can mimic pathologic abnormalities (Fig. 9) [50, 51]. Therefore, it is advised for non-specialised centres to ask assistance from experienced radiologists.

To conclude, post-mortem radiology without clinical autopsy is yet considered as insufficient to establish the cause of death, due to the low GRADE level of evidence. Autopsy is therefore still regarded as the reference standard [23]. Post-mortem radiology, especially as part of a MIA procedure, is considered a useful adjunct or valuable alternative in cases where autopsy is not performed. In general, neonatologists or paediatricians will be the referring physicians and as such they will be the ones obtaining parental informed consent. Therefore, it is imperative that they are aware of the advantages and limitations of post-mortem imaging. A multidisciplinary approach including clinicians, radiologists, and pathologists seems most beneficial. At present, PMMRI is the imaging modality of choice in foetuses, neonates, and young children, whereas PMCT is preferred in in older children.

## Appendix 1. Materials and methods

*Text adjusted from Acta Orthopedica (Besselaar et al *[52]; *PubMed PMID: 28266239)*.

### Guideline working group

This guideline was developed and sponsored by the Radiological Society of the Netherlands (NVvR), using governmental funding from the Stichting Kwaliteitsgelden Medisch Specialisten in the Netherlands (SKMS, Quality foundation of the Dutch Federation of Medical Specialists). The early preparative phase started July 2015 and the guideline will officially be authorized by the Radiological Society of the Netherlands at the end of 2017. The working group had nine in-person meetings (between September 2015 and September 2017) and otherwise communicated by phone and email. Decisions were made by consensus. At the start of guideline development, all working group members completed conflict of interest forms.

### Target group and aims

This guideline was developed for Dutch radiologists concerned with postmortem radiology, and other medical specialists involved in postmortem diagnostics. The main purpose of the guideline is to provide best possible care to fetuses or neonates, children and their relatives in a postmortem radiology setting, by informing optimal treatment decisions, and reduce unwarranted variation in the delivery of postmortem diagnostic care.

### Methodology

The guideline was developed in agreement with the criteria set by the advisory committee on guideline development of the Federation of Medical Specialists in the Netherlands (Medisch Specialistische Richtlijnen 2.0; OMS [53], which are based on the AGREE II instrument (Brouwers [54]; www.agreetrust.org). The guideline was developed using an evidence-based approach endorsing GRADE methodology, and meeting all criteria of AGREE-II. Grading of Recommendations Assessment, Development and Evaluation (GRADE) is a systematic approach for synthesizing evidence and grading of recommendations offering transparency at each stage of the guideline development [55, 56].

The guideline development process involves a number of phases, a preparative phase, development phase, commentary phase, and authorization phase. After authorization, the guideline has to be disseminated and implemented, and uptake and use have to be evaluated. Finally, the guideline has to be kept up-to-date. Each phase involves a number of practical steps (see Schünemann [57]).

A methodologist together with the chairman of the working group drafted a concept list of key issues which was extensively discussed in the working group. The selected (high priority) issues were translated into carefully formulated clinical questions, defining patient/problem, intervention, and prioritizing the outcomes relevant for decision-making. Particular attention was paid to relevant outcomes for relatives of fetuses or children undergoing postmortem radiology and defining minimal clinically important differences. Therefore, a focus group was organized in cooperation with the Federation of Patient Organizations in the Netherlands.

The literature was systematically searched using the databases MEDLINE (Ovid) and Embase (a detailed search strategy is available upon request). Selection of the relevant literature was based on predefined inclusion and exclusion criteria and was carried out by members of the working group (JE, RR) in collaboration with the methodologist (EK). For each of the clinical questions, the evidence was summarized by the guideline methodologist using the GRADE approach: a systematic review was performed for each of the relevant outcomes and the quality of evidence was assessed in one of four grades (high, moderate, low, very low) by analyzing limitations in study design or execution (risk of bias), inconsistency of results, indirectness of evidence, imprecision, and publication bias. The evidence synthesis was complemented by a working group member (JE or RR) considering any additional arguments relevant to the clinical question, including relatives values and preferences, and resource use (costs, organization of care issues). Evidence synthesis, complementary arguments, and concept recommendations were extensively discussed in the working group and final recommendations were formulated. Final recommendations are based on the balance of desirable and undesirable outcomes, the quality of the body of evidence across all relevant outcomes, values and preferences, and resource use. The strength of a recommendation reflects the extent to which the guideline panel was confident that desirable effects of the intervention outweigh undesirable effects, or vice versa, across the range of patients for whom the recommendation is intended. The strength of a recommendation is determined by weighting all relevant arguments together, the weight of the body of evidence from the systematic literature analysis, as well as the weight of all complementary arguments. Guideline panels must use judgment in integrating these factors to make a strong or weak recommendation. Thus, a low quality of the body of evidence from the systematic literature analysis does not exclude a strong recommendation, and weak recommendations may follow from high quality evidence [56].

After reaching consensus in the working group, the concept guideline was subjected to peer review by all relevant stakeholders. Amendments were made and agreed upon by the working group, and the final text was presented to the Dutch societies of medical specialists and other organizations that participated in the working group for approval and formal authorization. The guideline will be published and be freely accessible in the Dutch guideline database (Richtlijnendatabase, www.richtlijnendatabase.nl). The Dutch guideline database has a modular structure, with each clinical question as a separate entry, thus allowing for modular updates.

## Abbreviations

CR: Conventional radiography
GRADE: Grading Recommendations Assessment, Development and Evaluation
MaRIAS: Magnetic Resonance Imaging Autopsy Study
MIA: Minimally invasive autopsy
PMCT: Post-mortem computed tomography
PMMRI: Post-mortem magnetic resonance imaging
